# Hepatitis B and C viral infections among blood donors. A retrospective study from a rural community of Ghana

**DOI:** 10.1186/1756-0500-4-529

**Published:** 2011-12-12

**Authors:** Bernard Nkrumah, Michael Owusu, Paul Averu

**Affiliations:** 1 Kumasi Centre for Collaborative Research in Tropical Medicine, Kumasi, Ghana; 2 Komfo Anokye Teaching Hospital, Kumasi, Ghana; 3 Agogo Presbyterian Hospital, Asanti Akim North, Asanti Region, Ghana

## Abstract

**Background:**

Infection by Hepatitis B virus (HBV) and Hepatitis C virus (HCV) cause serious mortality, morbidity and financial burden and are thus a major global health problem. The study was conducted to investigate the prevalence of Hepatitis B and C infections and co-infections among blood donors in a rural community of Ghana.

This was a retrospective study conducted at the Agogo Presbyterian Hospital in the Asanti Akim North District of Ghana to investigate the prevalence of these infections over a three year period among 2773 blood donors. Males constituted a larger proportion of the study population (92.2%). Majority of the study population (43.9%) were within 26-35 age group. The disease prevalence was calculated at a 95% confidence interval.

**Findings:**

The prevalence of Hepatitis B viral (HBV) infection was highest in females- 21.4% (95% CI: 11.6-34.4) in 2006 than males in the same year- 13.2% (95% CI: 10.8-15.9). Hepatitis C viral (HCV) infection was highest among males- 11.6% (95% CI: 9.5-13.8) in 2007. HBV and HCV co-infection was higher in males- 2.6% (95% CI: 1.6-3.8) than females- 1.3% (95% CI: 0-7.0) in 2007. The overall prevalence of HBV and HCV was 13.8% (95% CI: 11.4- 16.4) and 9.4% (95% CI: 7.4-11.6) respectively in 2006. The rate of co-infection of HBV and HCV however increased from 1.6% (95% CI: 0.8-2.7) in 2006 to 2.2% (95% CI: 1.3-3.2) in 2008 in males and from 0% (95% CI: 0-6.4) in 2006 to 1.2% (95% CI: 0-6.5) in 2008 in females.

**Conclusion:**

The single infections of HBV and HCV reduced but co-infection of these transfusion transmitted infections increased. Measures such as more sensitive techniques for effective diagnosis and sanitary education to enlighten the population must be implemented.

## Introduction

Hepatitis B is one of most common infectious diseases of the world infecting two billion people including an estimated 400 million chronically infected cases [1]. Individuals with chronic infection have a high risk of developing liver cirrhosis and hepatocellular carcinoma. Hepatitis C virus infection is another common chronic blood borne infection with an estimated 3.9 million persons infected with the virus and have a high rate of development of liver cirrhosis. Infection by Hepatitis B virus (HBV) and Hepatitis C virus (HCV) cause serious mortality, morbidity and financial burden and are thus a major global health problem [2]. Few studies have previously indicated the high prevalence of HBV in Ghana [3,4] and predonation screening of blood donors or screening of donated blood for HBV and HCV are thus a routine practice. HCV is recognized as the primary cause of transfusion-associated non-A-non-B viral hepatitis worldwide [5] and is endemic in West Africa [6]. In 1996, Martinson and his group conducted a seroepidemiological survey of Hepatitis B and C virus infections in children attending school in Ashanti-Akim North district and showed that the overall HBV and HCV seroprevalence was 15.8% and 5.4% respectively [4] but recent studies conducted among blood donors in Ethiopia has shown decreasing trends of HBV and HCV [7]. From the Biostatistics department of the hospital, the prevalence of HBV and HCV in 2007 stood at 15.1% and 7.9% respectively (unpublished data).

Information on HBV and HCV seroprevalence in Ghana are old, scanty or limited to only urban blood donors. The study aim was therefore to determine the prevalence of HBV and HCV in blood donors in a rural area over a three year period (2006-2008).

## Methods

### Study site

The Agogo Presbyterian Hospital is located in the Asanti Akim North District of the Ashanti Region of Ghana, West Africa; and is the principal hospital of the district. The District is located in the eastern part of Ashanti Region and covers a land area of 1,160 km^2 ^with an estimated population of 142,434 (projection from 2000 Population Census). The catchment population of the hospital encompasses around 70,000 people, about 25,000 in Agogo itself. Agogo is surrounded by hills covered with tropical secondary forests, subsistence and small commercial acreages, grass and bush land that had emerged after forest clearing due to logging and farming activities. The most important cash crops are cocoa and oil palm. Bananas, plantains, yams, cassava and maize are the most important crops for subsistence farming.

### Study population

This consisted of voluntary and replacement donors who presented to the blood bank of the hospital. Between January 2006 and December 2008, serological screening results from 2773 blood donors were obtained from the hospital's archive. Prospective donors between the ages of 17-60 years who pass a history screen of their medical and physical state and predonation screening test for Hb level, HBV, HCV, Syphilis and HIV were considered fit to donate blood.

### Sampling

The screening of blood donors or donated blood for HBsAg and anti-HCV is mandatory thus blood donations from individuals who are found to be positive for any of the above infections were not done. Archived results from the hospitals blood bank was used for this study with approval from the hospital authorities. The donor samples were tested using the DiaSpot^® ^One Step Hepatitis test kits (DiaSpot Rapid Diagnosis, Pondok Kelapa 13450, Jakarta Indonesia). The tests were carried out according to the manufacturer's instructions.

### Quality control

In house positive and negative controls were performed for each reagent lot.

### Statistical analysis

Data were double-entered into a predesigned electronic database using Epi info version 6.04dfr (Center for Disease Control, Atlanta, GA, USA) and cleaned. Data was exported to *Stata/*SE11.1 statistical software (Stata Corporation, Texas USA) for analysis. The prevalence was calculated at a 95% confidence interval.

### Ethical Approval

Ethical approval for the study was obtained from the Committee on Human Research, Publication and Ethics (CHRPE), of the School of Medical Sciences, KNUST-Kumasi.

## Results

**Figure 1:**
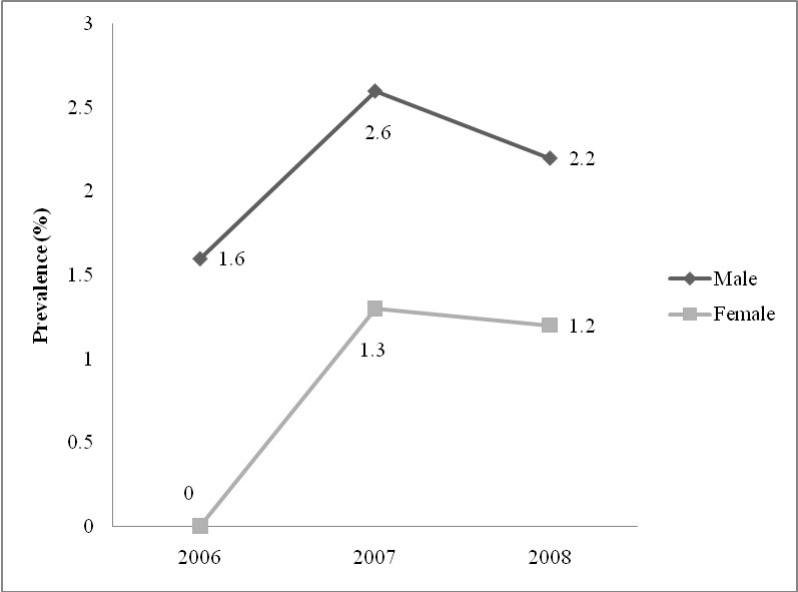
**HBV and HCV co-infection among males and females**.

**Figure 2:**
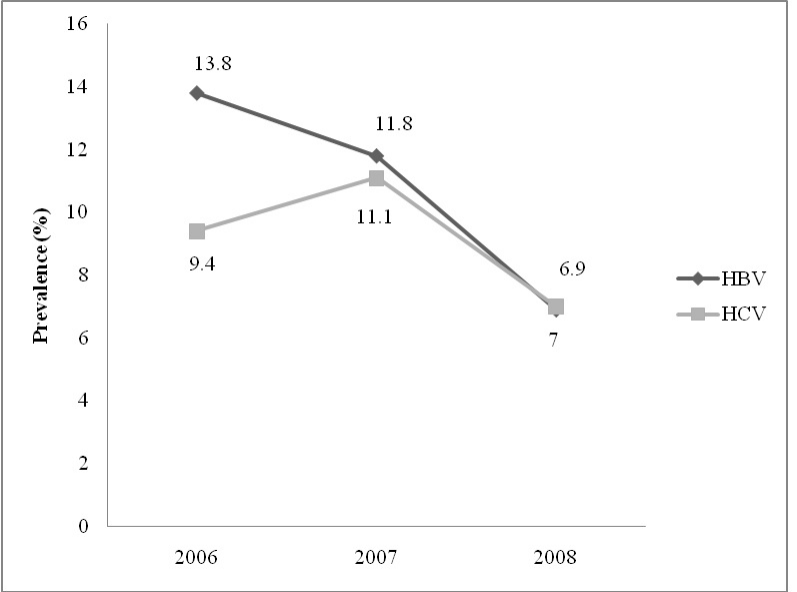
**Overall prevalence of HBV and HCV infection**.

**Figure 3:**
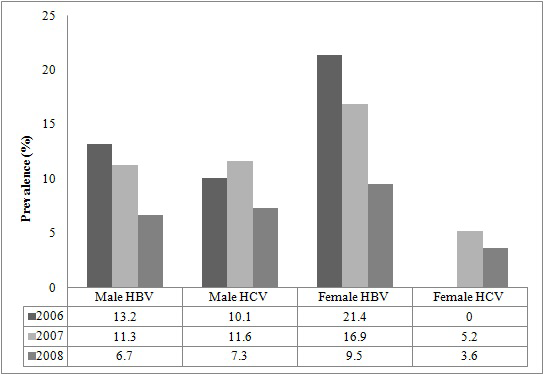
**Prevalence of HBV and HCV infection among males and females**.

A total of 2773 prospective blood donors were screened from January 2006 to December 2008. Study participants were grouped into five age categories (Table 1). Out of these, 2556 (92.2%) were males and 217 (7.8%) were females. Donors were categorized into five age groups. The majority of the study population- 1217 (43.9%) - were within the 26-35 age group. Analysis of the prevalence of HBV and HCV among males and females revealed a statistically significant decrease in the occurrence of the infections among males and females (Table 2). Of the total number, 10.53% (292/2773), 5.63% (156/2773) and 2.09% (58/2773) were HBV, HCV and both HBV and HCV positive, respectively. Furthermore, the prevalence of co-infection of HBV and HCV decreased for the years under review with the highest rate peaking at 2.6% (95% CI: 1.6-3.6) in 2007 (Figure 1). The overall prevalence rate for HBV was highest in 2006 (13.8%; 95% CI: 11.4-16.4) but decreased in 2008 to 6.9% (95% CI: 5.4-8.6) (Figure 2). The overall prevalence of HCV was highest in 2007 (11.1%; 95% CI: 9.2-13.2) but decreased to 7.0% (95% CI: 5.5-8.8) in 2008. The prevalence rate of HBV was relatively higher in females but vice versa for HCV (Figure 3).

**Table 1:** Age categories of the study population

	2006		2007		2008		Total
	
Age Group	Male	Female	Male	Female	Male	Female	
16-25	194	25	198	37	254	49	757
26-35	297	15	450	21	413	21	1217
36-45	152	5	183	13	203	9	565
46-55	64	5	61	4	61	5	200
56-65	7	6	8	2	11	0	34

**Table 2:** Co-infection of HBV/HCV from 2006 to 2008

	Prevalence at 95% CI								
	
	HBV only		HCV only		Both HBV and HCV		Overall Prevalence		
	
Year	Male	Female	Male	Female	Male	Female	HBV	HCV	Both HBV and HCV
2006	13.2^†^	21.4	10.1	0	1.6	0	13.8	9.4	1.6
	(10.8-15.9)*	(11.6-34.4)	(8.0-12.5)	(0-6.4)	(0.9-2.9)	(0-6.4)	(11.4-16.4)	(7.4-11.6)	(0.8-2.7)

2007	11.3	16.9	11.6	5.2	2.6	1.3	11.8	11.1	2.6
	(9.3-13.6)	(9.3-27.1)	(9.5-13.8)	(1.4-12.8)	(1.6-3.8)	(0-7.0)	(9.8-14.0)	(9.2-13.2)	(1.6-3.6)

2008	6.7	9.5	7.3	3.6	2.2	1.2	6.9	7	2.2
	(5.2-8.5)	(4.2-17.9)	(5.7-9.2)	(0.7-10.1)	(1.4-3.4)	(0-6.5)	(5.4-8.6)	(5.5-8.8)	(1.3-3.2)

† Percentage prevalence

* Confidence interval

## Discussion

HBV and HCV infections occurrence among blood donors in a rural setting was determined by serological methods and the results were compared to assess the trends in three consecutive years, 2006, 2007 and 2008. The prevalence of viral carrier rates in the blood donors appears to be different to that of urban blood donors as shown in the data with a decrease in HBV and HCV. Statistics from earlier studies showed high rates of HBV and HCV in urban blood donors [7-9]. Simultaneous increase of HBV and HCV infections might have been caused by sexual transmission through unprotected sex and other practices [10-12]. Our study raises serious concerns regarding the safety of the blood supply in our rural communities where resources are hard to come by. The decreasing rate of positivity to HBV and HCV or both suggests that horizontal rather than vertical transmission is the major source of this endemicity [10]. In areas of low endemicity, transmission occurs primarily among young adults [13] and there is an age effect on the prevalence of HBV and HCV infections [14]. Horizontal transmission of HBV and HCV have been related to age, socioeconomic conditions, socio professional status and risky behaviors such as sharing of bath towels, chewing gum, partially eaten candies, or dental cleaning materials, as well as biting fingernails in conjunction with scratching the backs of carriers [15-17]. It has been shown that the improvement of socioeconomic conditions may lead to a decreasing exposure to HBV and HCV infections [17] thus an increased risk of HBV and HCV infections might be related to an increased exposure to risk factors in conjunction with poor sanitary and socioeconomic conditions. The decreasing trend of HBV and HCV infections in our study population might be due to decreased exposure to risk factors in conjunction with improving sanitary and socioeconomic conditions. The fact that our study community is being educated through weekly radio health talk programmes on these diseases in terms of good life style practices such as having protected sex, not sharing razors and needles with other people among others are been adhered to. Another possible reason might be the fact that screening of blood donors for HBsAg and anti-HCV does not totally eliminate the risk of HBV and HCV infection through blood transfusion since donors with occult HBV and HCV infection which lacked detectable levels of HBsAg and anti-HCV [18] were screened as negative. This emphasizes the need for a more sensitive and stringent screening algorithm for blood donations even in rural settings.

## Conclusion

HBV and/or HCV infection(s) among blood donors in the study area is/are reducing. The occurrence of these infections among the blood donors should still be monitored carefully to further reduce the rates to ensure safer and more reliable blood for transfusion. Measures such as more sensitive techniques, education, sensitization and vaccination must be carried out to ensure that people are well enlightened and protected from these infections.

## Competing interests

The authors declare that they have no competing interests.

## Authors' contributions

BN designed the study protocol, analyzed and interpreted the data and headed the writing of the protocol. MO contributed to the writing of the manuscript. PA headed the laboratory team and contributed to the writing of the manuscript. All authors have read and approved the final version of this manuscript.
